# Reply to Pastore, E.P. Comment on “Rastogi et al. Brain Tumor Detection and Prediction in MRI Images Utilizing a Fine-Tuned Transfer Learning Model Integrated Within Deep Learning Frameworks. *Life* 2025, *15*, 327”

**DOI:** 10.3390/life16040536

**Published:** 2026-03-24

**Authors:** Deependra Rastogi, Prashant Johri, Massimo Donelli, Lalit Kumar, Shantanu Bindewari, Abhinav Raghav, Sunil Kumar Khatri

**Affiliations:** 1 School of Computer Science and Engineering, IILM University, Greater Noida 201306, India; 2 School of Computing Science and Engineering, Galgotias University, Greater Noida 203201, India; 3 Department of Civil, Environmental, Mechanical Engineering University of Trento, 38100 Trento, Italy; 4 Radiomics Laboratory, Department of Economy and Management, University of Trento, 38100 Trento, Italy; 5 Amity University, Noida 201313, India

We are grateful to Dr. Pastore for his thoughtful comments [1] on our article. Below, we provide a revised and carefully edited response addressing the points raised.

We appreciate the reviewer’s insightful observation regarding the potential limitations associated with dataset selection and experimental design. The primary objective of our study was to conduct a systematic and controlled comparison of multiple fine-tuned transfer-learning architectures for brain tumor classification, with an emphasis on methodological benchmarking rather than immediate clinical deployment. To this end, we employed a publicly available Kaggle-sourced MRI dataset, which facilitates transparency, reproducibility, and direct comparison with prior studies, as recommended in early-stage deep learning research for medical imaging [2].

We sincerely thank the reviewer for highlighting the critical issue of subject-level data separation, which is widely recognized in the medical imaging literature as a prerequisite for valid model evaluation. Brain MRI datasets frequently consist of multiple highly correlated slices per subject, often acquired across different sequences and imaging parameters. When random image-level splitting is employed, there is a substantial risk that anatomically adjacent or near-duplicate slices from the same individual may appear in both training and testing sets. This scenario can lead to inadvertent information leakage, enabling models to leverage subject-specific anatomical cues, scanner-dependent textures, or acquisition artifacts rather than learning disease-specific and generalizable representations [3,4]. In the present study, we relied on a publicly available Kaggle MRI dataset that did not include explicit patient, sequence, or site identifiers, thereby limiting our ability to implement strict patient-level or sequence-aware splitting. This constraint reflects a known limitation of several open-access imaging repositories frequently used for benchmarking purposes. To partially mitigate redundancy effects, we confined data augmentation strictly to the training set and ensured that no augmented samples were shared across partitions. However, we fully acknowledge that these measures cannot fully replace subject-level separation and that residual optimistic bias may remain in the reported performance.

We appreciate the reviewer’s detailed and constructive feedback regarding the risk of pipeline leakage arising from improperly nested preprocessing, augmentation, and model optimization steps. We fully agree that all data-dependent operations must be strictly confined to the training folds within a resampling framework to ensure unbiased estimation of model performance. The medical imaging literature has repeatedly emphasized that failure to nest these steps appropriately can introduce subtle but significant information leakage, leading to inflated discrimination metrics and reduced reproducibility [5]. Preprocessing operations such as intensity normalization, resizing, and imputation inherently encode statistical properties of the data distribution. When these transformations are estimated using the full dataset—rather than exclusively on training partitions—information from the held-out data can inadvertently influence the learned feature space, violating the independence of evaluation sets [4]. Similarly, data augmentation strategies, while essential for improving generalization in deep learning models, must be applied only to training samples; applying augmentation prior to data splitting or across folds risks introducing correlated samples into validation or test sets, thereby biasing performance estimates [3,4]. In the present study, all preprocessing and augmentation procedures were implemented using the training data only, and the resulting transformations were applied unchanged to the validation and test sets. Augmentation was performed exclusively on the training subset to avoid redundancy-induced bias. Hyperparameter tuning and fine-tuning schedules were optimized on a dedicated validation split and were not adjusted based on test-set performance. The final evaluation was conducted on a held-out test set that remained untouched throughout model development, thereby preserving the integrity of performance estimation.

We sincerely thank the reviewer for highlighting this important consideration. We agree that evaluating model transportability under data drift and varying acquisition conditions is essential for assessing clinical robustness [6]. In this study, we focused on a single train–validation–test division to establish a baseline comparison across transfer-learning architectures. We acknowledge that repeated nested cross-validation, inclusion of temporally or externally separated test sets, and calibration analyses (e.g., reliability diagrams, decision curve analysis) would provide a stronger assessment of generalization and clinical utility. These aspects will be prioritized in future work.

## Data Availability

Data will be made available on “Brain tumor [WWW Document], 2020. Kaggle. https://www.kaggle.com/datasets/jakeshbohaju/brain-tumor, accessed on 16 February 2025”.
